# Unveiling the Degradation Mechanism of Impermeability and Pore Structure in Concrete Under Long-Term Water Exposure

**DOI:** 10.3390/ma19030496

**Published:** 2026-01-26

**Authors:** Hua Wei, Yi Sun, Chunhe Li, Yang He, Hao Lu, Lan Tang

**Affiliations:** 1 Materials & Structural Engineering Department, Nanjing Hydraulic Research Institutes, Nanjing 210029, China; hwei@nhri.cn (H.W.); tanglano@163.com (L.T.); 2 Dam Safety Management Department, Nanjing Hydraulic Research Institute, Nanjing 210029, China; sunyihhu@163.com; 3 Dam Safety Management Center of the Ministry of Water Resources, Nanjing 210029, China; 4 Civil and Environmental Engineering, The University of Miyazaki, 1-1 Gakuenkibanadainishi, Miyazaki 889-2192, Japan; lichunhe@cc.miyazaki-u.ac.jp

**Keywords:** concrete impermeability, pore structure, hydration composition, phase analysis

## Abstract

To investigate the evolution of impermeability and pore structure in concrete under long-term service, systematic tests were conducted on submerged concrete from dam sections with over 75 years of service. Results show that the surface water permeation resistance index of concrete in the downstream section of the main dam is only 9.19 × 10^−9^, significantly lower than that of concrete from the upstream of the main dam (UMD), downstream of the main dam (DMD), upstream of the auxiliary dam (UAD), and upstream of the weir (UW). Moreover, its impermeability improves noticeably within the 0–100 mm depth range. Mercury intrusion porosimetry revealed that the median pore diameter, average pore diameter, pore content, and porosity in this region reach 306.7 nm, 55.4 nm, 35.64%, and 3.961 mm, respectively—all markedly higher than in other dam sections. Combined XRD and SEM/EDS analyses indicate that crystallization pressure from saline solutions in the coastal environment, together with long-term carbonation, leads to structural loosening and increased porosity in the downstream concrete of the main dam, ultimately degrading its impermeability performance.

## 1. Introduction

As the core material in modern hydraulic engineering structures, the long-term durability of concrete directly impacts the safety and service life of major infrastructure such as dams, cross-sea bridges, port terminals, and undersea tunnels [1,2,3]. Throughout their service life, these structures are subjected to relentless challenges from multiple coupled factors, including hydraulic gradients, the permeation of aggressive ions, wet–dry cycles, freeze–thaw cycles, and scouring by water flow [3,4]. Among these, impermeability serves as the primary barrier in concrete’s durability system. It fundamentally determines the ease with which external harmful agents, such as chloride and sulfate ions, penetrate the concrete matrix, thereby influencing the initiation and progression of reinforcement corrosion, chemical attack, and freeze–thaw damage. However, despite extensive research on concrete durability, the evolution mechanisms of impermeability and pore structure under long-term water exposure remain insufficiently understood. Specifically, it is still unclear how microstructural changes, including porosity, pore size distribution, and pore connectivity, affect macro-scale impermeability over decades of service, particularly under the simultaneous action of multiple aggressive factors [5]. Therefore, investigating the evolution patterns and mechanisms of impermeability and pore structure in concrete under long-term water exposure, which has become a critical scientific issue for ensuring the safety of major engineering projects throughout their lifecycle and advancing the development of long-life design theories [6].

The impermeability of concrete is intrinsically linked to its internal micro-pore structure. As a typical porous and heterogeneous composite, the permeability of concrete is predominantly controlled by its porosity, pore size distribution, pore connectivity, and the properties of the interfacial transition zone between the cement paste and aggregates [7,8,9,10]. Research has shown that the pore structure of cementitious materials undergoes dynamic evolution [11,12]. In aggressive aqueous environments, such as those containing chloride or magnesium ions, complex physicochemical interactions with cement hydration products can initially induce temporary pore refinement due to the formation of expansive products. However, with prolonged exposure, ongoing dissolution and ion exchange reactions accelerate the degradation of the interfacial transition zone, resulting in pore coarsening and the development of more interconnected permeation networks [13]. Wang Yuanzhan from Tianjin University conducted a comprehensive analysis of chloride ion transport in marine concrete environments. The study encompassed chloride transport models, concrete composition, water level fluctuation zones, the presence of sulfate and magnesium ions, freeze–thaw cycles, loading effects, and concrete anti-corrosion coatings, including their resistance to chloride-induced degradation [14]. Gu Linan from Dalian Jiaotong University investigated the effects of seawater curing on the structure and properties of concrete containing different mineral admixtures. Mechanical property studies revealed that seawater curing enhances early compressive strength but reduces long-term strength. Mercury porosimetry indicated that seawater curing reduces concrete porosity, while mineral admixtures refine the pore structure. It mitigates the detrimental effects of harmful ions in seawater on concrete structure. Among the tested admixtures, silica fume outperformed fly ash and a 1:1 blend of mineral powder with zeolite powder. The optimal dosage of silica fume was determined to be 8% [15]. Microscopic investigations of concrete exposed to seawater reveal a markedly degraded pore structure and interfacial transition zones compared with freshwater-exposed specimens, characterized by enlarged pores, increased cracking, and reduced microhardness within the transition zones. In cold regions, alternating freeze–thaw cycles combined with abrasion synergistically compromise concrete at both macro- and micro-scales, further accelerating pore structure deterioration and reducing permeability [16,17,18,19,20]. Although extensive research has been conducted on the effects of water and seawater on concrete structural properties [1,16,17], accurately characterizing and quantifying the evolution of concrete pore structure under long-term [7,8,13], multi-factor coupled influences—and establishing a predictive correlation with macro-scale impermeability—remains a major challenge in this field. Existing studies have shown that pore structure evolution is governed by the combined effects of chemical interactions (e.g., chloride, sulfate, and magnesium attack), physical damage (e.g., freeze–thaw cycles and erosion), and mechanical loading, leading to highly nonlinear and spatially heterogeneous degradation processes [5,9,10,13,21]. Moreover, most current investigations focus on isolated microstructural indicators or short-term observations, while robust predictive correlations linking pore structure parameters to macro-scale impermeability over long service periods are still lacking [7,8,13,22].

Therefore, this study focuses on investigating the water resistance and pore structure of concrete exposed to water under long-term service conditions. Concrete surface erosion and macroscopic water resistance are analyzed and evaluated, while mercury intrusion porosimetry (MIP) and bubble parameter testing are employed to characterize structural features at different locations within water-exposed environments, revealing their interrelationship. Mechanisms of material transformation at the chemical and elemental levels are further examined using X-ray diffraction (XRD), scanning electron microscopy (SEM), and energy-dispersive spectroscopy (EDS). By integrating pore structure analysis with studies of material reaction deposits, this approach aims to elucidate the underlying mechanisms governing permeability performance.

## 2. Material Proportions and Test Methods

### 2.1. Material Proportions

**Figure 1:**
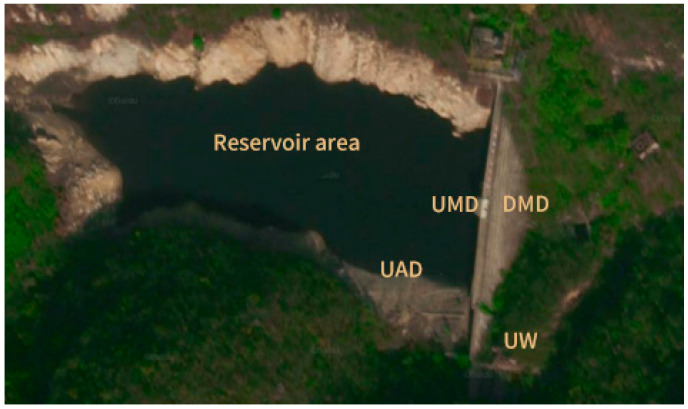
The schematic location of UMD; DMD; UAD; UW.

**Figure 2:**
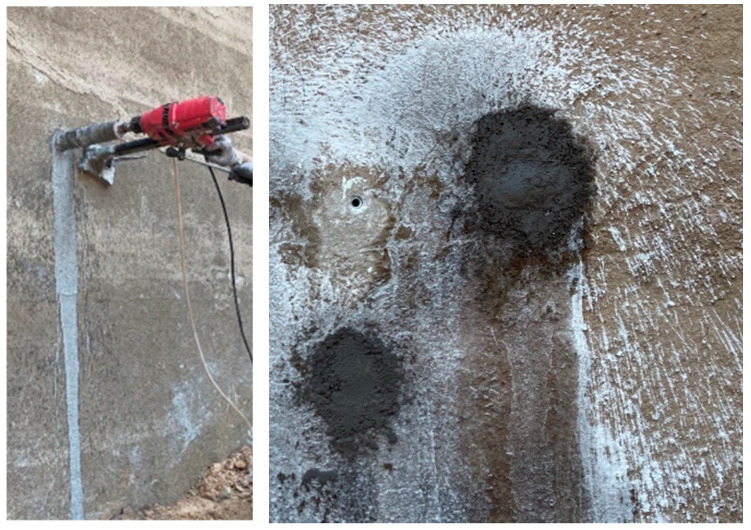
Dam sampling and sealing process.

A reservoir in Lianyungang City, Jiangsu Province, has been in operation for 78 years and has been classified as a Class III dam during its operational period. Both the main and auxiliary dams are concrete gravity dams. The upstream dam surface has an elevation of 198.70 m, with a designed storage capacity of 336,000 m^3^ and a corresponding design flood level of 205.60 m. A weir is located on the right side of the auxiliary dam, and the overflow weir has a clear width of 21.90 m, as shown in Figure 1. In situ concrete samples were obtained from its upstream and downstream water-facing surfaces, dam sampling and sealing process are shown in Figure 2. Primary testing locations include UMD, DMD, UAD, UW. In view of the long service life of the structures and the limitations of concrete technology at the time, this study presents the typical concrete mix proportions in Table 1, aiming to provide a reference for analyzing performance differences and pore structure evolution in concrete from different locations. Concrete types tested comprise two-graded and three-graded mixes, with the upstream faces of the main dam and auxiliary dam, along with the upstream face of the weir structure, utilizing two-graded concrete, while the upstream face of the auxiliary dam employs three-graded concrete.

### 2.2. Analysis of Relative Permeability Coefficient Testing

**Figure 3:**
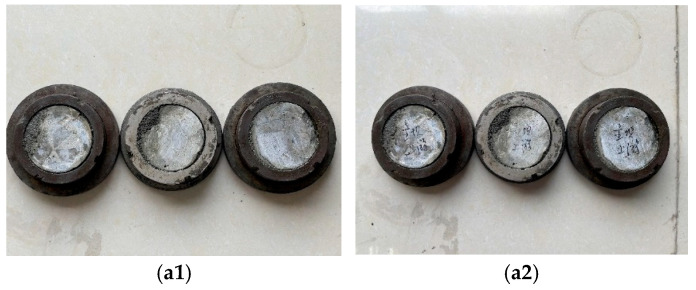

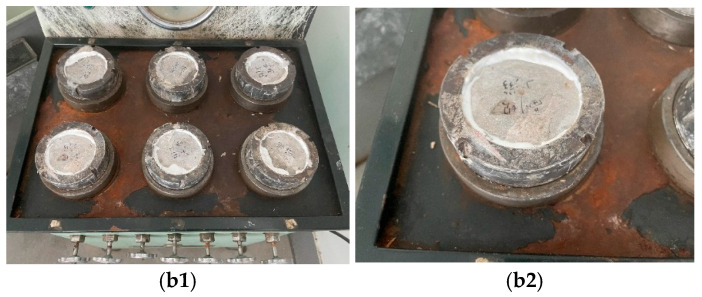
Permeability test process: (**a1**,**a2**) represents specimen molding, (**b1**,**b2**) represents permeability testing.

In accordance with the Code for Testing Hydraulic Concrete (SL/T 352-2020) [23], core samples were machined into cylinders with a diameter of 70 mm. Each cylinder was then sectioned perpendicular to its axis into three smaller cylindrical specimens, each 50 mm in height, representing dam concrete at three distinct depth zones: 0–50 mm, 50–100 mm, and 100–150 mm. The specimens were placed in mortar permeability test molds, with the side corresponding to the dam’s outer surface designated as the permeable face. The specimen has an upper diameter of 70 mm, a lower diameter of 80 mm, and a height of 30 mm. High-strength mortar was used to fill the molds, and the specimens were cured under standard conditions for 28 days. Following consolidation and hardening, the specimens were subjected to permeability testing. The water penetration resistance was comprehensively evaluated based on three indicators: whether penetration occurred, the penetration height [mm], and the permeability index. In cases where penetration led to test termination, the time to penetration [h] was recorded to account for early failure. The test permeation pressure was set at 81,600 mm (0.8 MPa), with a permeation duration of 24 h. Photographs of the specimen molding and testing process are shown in Figure 3:

### 2.3. Analysis of MIP Microporous Structure

Hardened paste samples were extracted from the surface layer, 50 mm depth, and 100 mm depth within the drilled cores. The pore structure of the 2.0 g concrete paste was characterized using an American Micro Instrument AutoPore IV 9500 (Micromeritics Instrument Corporation, Norcross, GA, USA) fully automated mercury intrusion porosimeter. Pore size measurements ranged from 50 Å to 1000 μm, with a maximum applied pressure of 228 MPa.

### 2.4. Pore Parameter Testing

**Figure 4:**
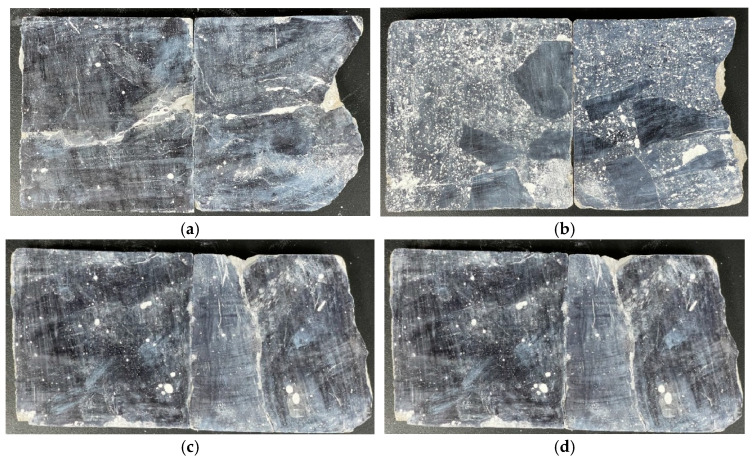
Preparation of hardened air-void specimens: (**a**) UMD; (**b**) DMD; (**c**) UAD; (**d**) UW.

The bubble parameter test method was conducted according to the Concrete Bubble Parameter Test (Straight Wire Method) [23,24]. Cylindrical core samples of concrete were cut along the diameter, polished to a mirror finish using a 1500-grit or finer abrasive wheel, and treated as required for hardened bubble parameter testing before experimentation. The treated samples are shown in Figure 4, where the chart depicts progressive penetration from the surface to the interior from left to right. The instrument used was the Rapid-air 3000 Sakura Concrete Pore Tester (Rapid-Air & MTE Hydraulics Co., Ltd., Rockford, IL, USA).

### 2.5. X-Ray Diffraction Phase Analysis

The chemical composition of the cavitation test specimens was characterized qualitatively by X-ray diffraction (XRD) using a SmartLab system (Rigaku Corporation, Tokyo, Japan). The XRD analysis was operated at a voltage of 45 kV and a current of 200 mA with a Cu target. The scanning parameters were set to a range of 5–80° at a speed of 10°/min.

### 2.6. Microstructural Testing

The pore structure of UWP samples at the micro-scale was characterized using SEM. Scanning electron microscopy primarily utilizes secondary electron signals to image sample surface morphology. A Helios Nanolab 600 SEM (FEI Company, Hillsboro, OR, USA)with an accelerating voltage of 5 kV and an emission current of 86 pA was used for microscopic morphology observation of fracture faces of the UWP specimens. Gold was coated on the sample surface before observation to mitigate charging.

## 3. Test Results and Analysis

### 3.1. Relative Permeability Test

**Figure 5:**
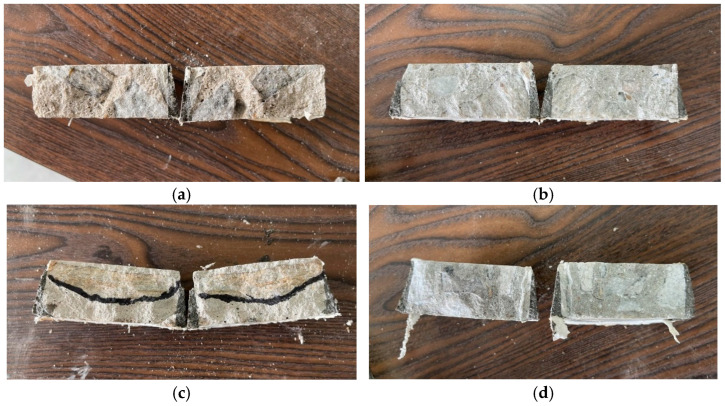
Permeability test results: (**a**) UMD, (**b**) UAD, (**c**) UW, (**d**) DMD.

Photographs of the permeation test results are shown in Figure 5. It can be observed that no permeation or water marks were present on UMD, nor on the upstream side of the auxiliary dam. No permeation was observed on the upstream side of the weir structure, but distinct water marks appeared. Meanwhile, significant water pressure permeation was evident on the DMD. No water marks appeared on UMD or UAD, but water marks did appear on the weir structure. This is because the flow conditions differ across the various dam structures: the concrete of the main dam and auxiliary dam is subjected to static water pressure, while the weir structure experiences erosion on its concrete surface due to the passage of flowing water. Furthermore, compared to the upstream face, the downstream face exhibited pronounced seepage. While it maintained some structural impermeability, its effectiveness was significantly reduced, warranting further investigation and analysis.

Permeability tests were conducted at different locations and depths. The experimental results are shown in Table 2. The permeability coefficient of concrete was calculated using the following Formula (1):

(1) $$D_{\mathrm{RCM}\;}=\;\frac{0.0239\;\times \;(273\;+\;T)L}{(U\;-\;2)t}(X_{d}\;-\;0.0238\sqrt{\frac{(273\;+\;T)LX_{d}}{U\;-\;2}})$$

In the equation, $D_{RCM}$ represents the non-steady-state chloride ion migration coefficient in pure paste; U denotes the absolute value of the applied voltage; T is the average of the initial and final temperatures of the anode solution, in °C; L is the specimen thickness, in mm; $X_{d\;}$ is the average chloride ion penetration depth, in mm; and t is the test duration, in hours.

Table 2 indicates that after 78 years of service, concrete specimens from the same location exhibit a noticeable decrease in permeation height across the 0–50 mm, 50–100 mm, and 100–150 mm depth ranges. In contrast, specimens from the downstream section of the main dam show a significant increase in permeation. These results suggest that durability issues have developed on the concrete surface, while the internal structure remains largely unaffected. The impermeability strength of concrete decreased significantly between 0–50 mm and 50–100 mm, while the increase in permeability performance for concrete structures between 100 mm–150 mm was markedly reduced. This indicates that the primary depth affecting the reduction in concrete water resistance strength is within the top 100 mm. To elucidate the underlying cause of this strength reduction, pore property tests were conducted.

### 3.2. Pore Structure Analysis

#### 3.2.1. Analysis of Mercury Injection Test Results

**Figure 6:**
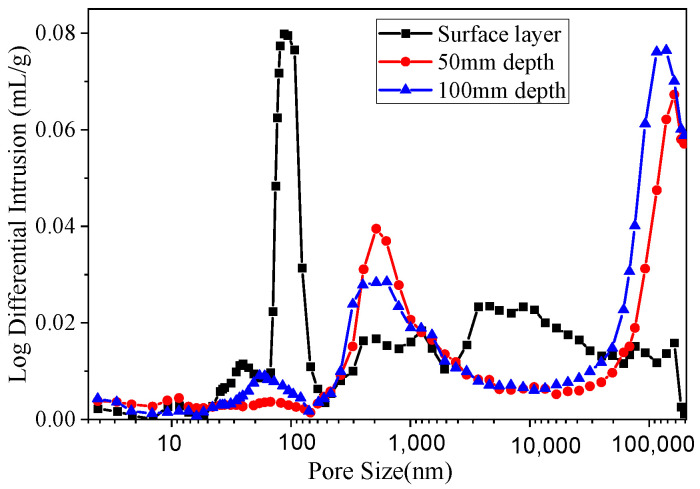
Concrete pore structure distribution.

Based on permeability test results, the section exhibiting the lowest overall impermeability is the downstream face of the main dam. Consequently, concrete samples from this section were collected at different depths corresponding to the impermeability test: three specimens representing the surface layer, 50 mm depth (middle layer), and 100 mm depth (inner layer) were selected. The internal pore structures of these specimens were subsequently analyzed using mercury intrusion porosimetry (MIP), as shown in Figure 6.

**Figure 7:**
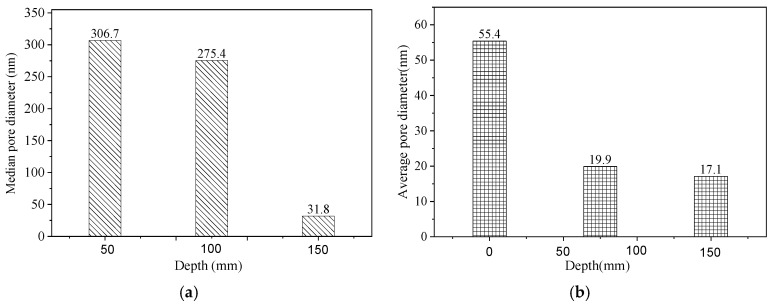
Pore diameter distribution in concrete: (**a**)—Median pore diameter; (**b**)—Average pore diameter.

This figure illustrates the pore size distribution pattern within the concrete of the main dam’s downstream seaward face. It is evident that the cumulative pore distribution within the concrete remains relatively consistent at depths of 0–30 mm and 30–60 mm. A distinct peak in pore structure distribution occurs around 900 nm, with another peak appearing at the 0.1 mm macropore size—likely attributable to external erosion damage. In contrast, the internal structure exhibits smaller and more uniform pores, with a significant distribution around 100 nm. Further computational analysis statistically evaluated the median and mean pore diameters at different depths within the concrete, as shown in Figure 7.

As shown in the figure, the median pore diameter of the surface-layer concrete is 306.7 nm, that of the middle-layer concrete is 275.4 nm, and that of the inner-layer concrete is 31.8 nm. The average pore diameter of the surface-layer concrete is 55.4 nm, compared with 19.9 nm for the middle layer and 17.1 nm for the inner layer. These data clearly indicate that the concrete surface layer facing the sea downstream of the main dam contains a higher number of large pores, with fewer large pores present in the inner layers. From the surface to the interior, the degree of compaction increases progressively. According to established pore size classifications and their performance impacts in fully hydrated cement, pores smaller than 10 nm are classified as gel pores, medium capillaries range from 10 to 50 nm, large capillaries from 50 nm to 1 μm, and pores larger than 1 μm are considered air-entrained pores. Gel pores primarily influence cement paste shrinkage and creep, medium capillaries affect paste strength, permeability, and shrinkage under high humidity, large capillaries mainly influence strength and permeability, and air-entrained pores predominantly affect cement paste strength. The pore structure analysis, as illustrated in the charts, shows that surface-layer concrete exhibits significantly higher pore volumes than the middle and inner layers at a pore size of 10 μm. These large pores substantially reduce concrete strength and permeability, classifying them as detrimental pores in concrete structures. This microscopic pore distribution mechanism explains the macroscopic permeability test results, specifically the lower impermeability observed in surface-layer concrete.

#### 3.2.2. Pore Parameter Analysis

**Figure 8:**
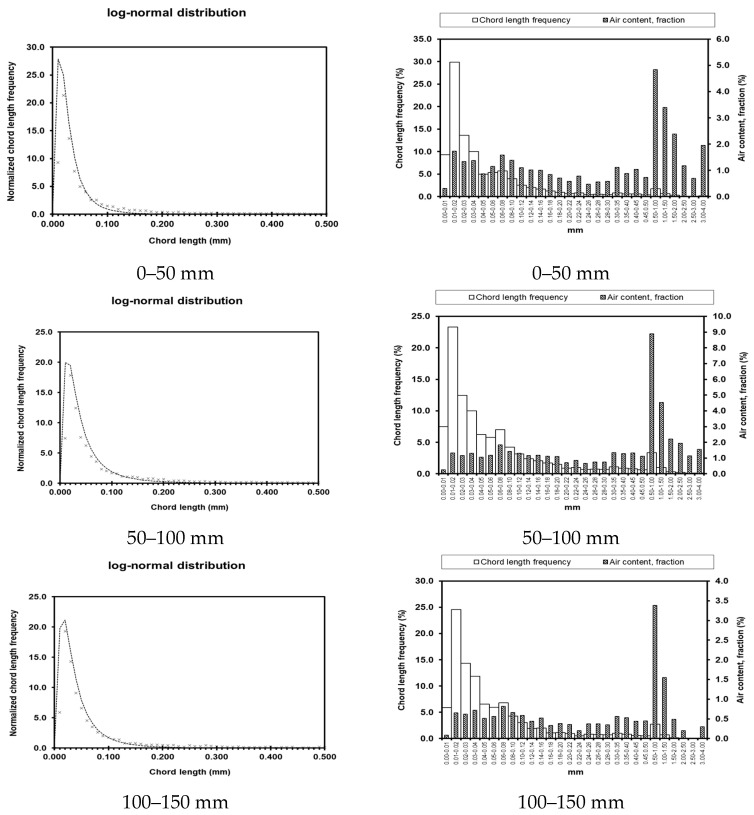
Pore parameters of UMD.

**Figure 9:**
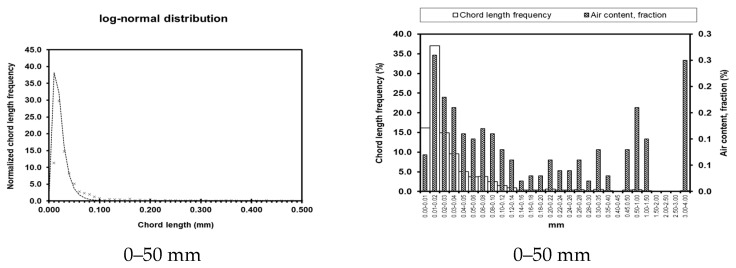

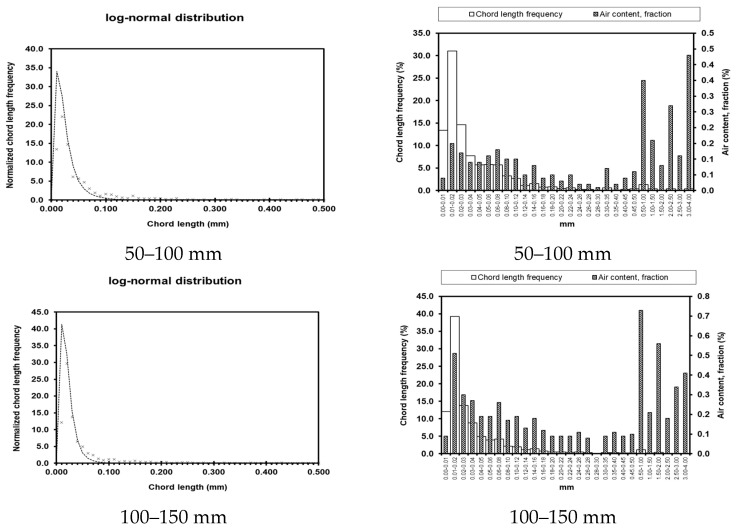
Pore parameters of DMD.

**Figure 10:**
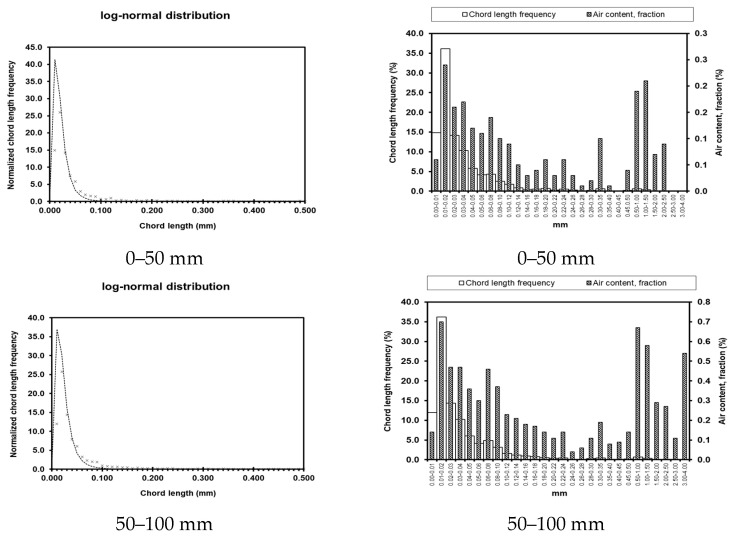

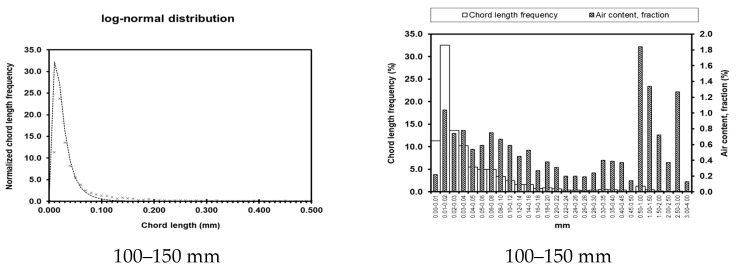
Pore parameters of UAD.

**Figure 11:**
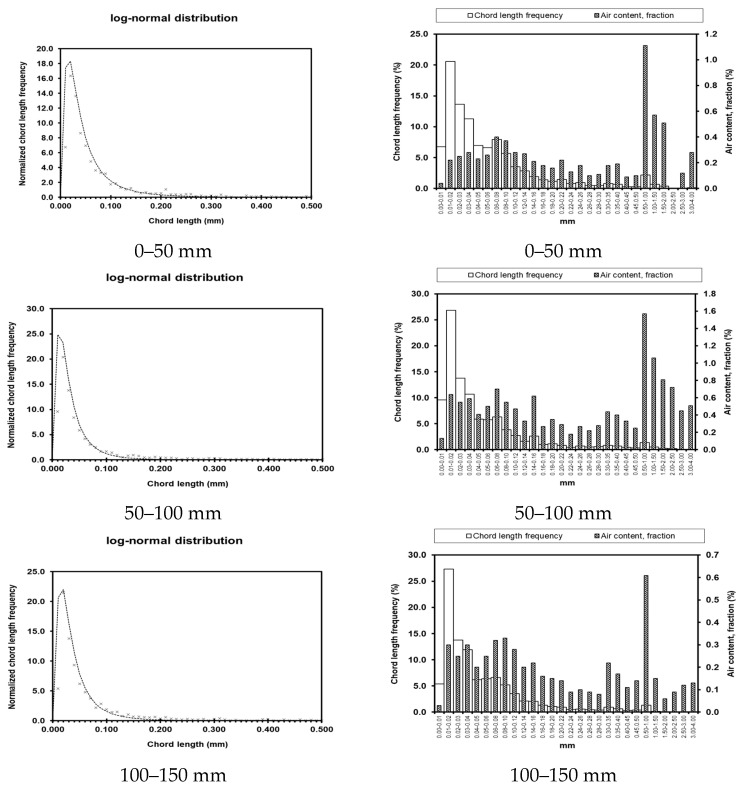
Porosity parameters of UW.

Test results for pore parameters at different locations and depths within the dam body are presented in Table 3. Graphical representations of these results are shown in Figure 8, Figure 9, Figure 10 and Figure 11. The left column shows the standardized chord length distribution, which characterizes the spatial spacing of air voids, while the right column presents the pore content distribution, representing the size distribution of the pores.

The bubble spacing coefficient represents the average distance from any point within the slurry to the nearest pore. At a given air content, smaller bubble sizes correspond to lower spacing coefficients. Extensive research indicates that bubble structures with smaller spacing coefficients have a reduced impact on concrete strength and contribute to enhanced durability. As summarized in Table 3, the measured pore parameters of concrete at different locations show that although the pore content and porosity coefficients of UMD and UAD exhibit slight fluctuations, they remain within reasonable limits. In contrast, the hardened concrete on the seaward face of DMD exhibits a high pore content of 35.64% and a porosity coefficient of 3.961 mm, corresponding to a bubble spacing coefficient of 0.0132. These values are significantly higher than those of other concrete sections, indicating substantially reduced compaction on the seaward face of the downstream main dam compared with the upstream face. The measured strength and impermeability results further corroborate these microstructural observations from a macro-performance perspective.

### 3.3. Component and Phase Analysis

#### 3.3.1. X-Ray Analysis

**Figure 12:**
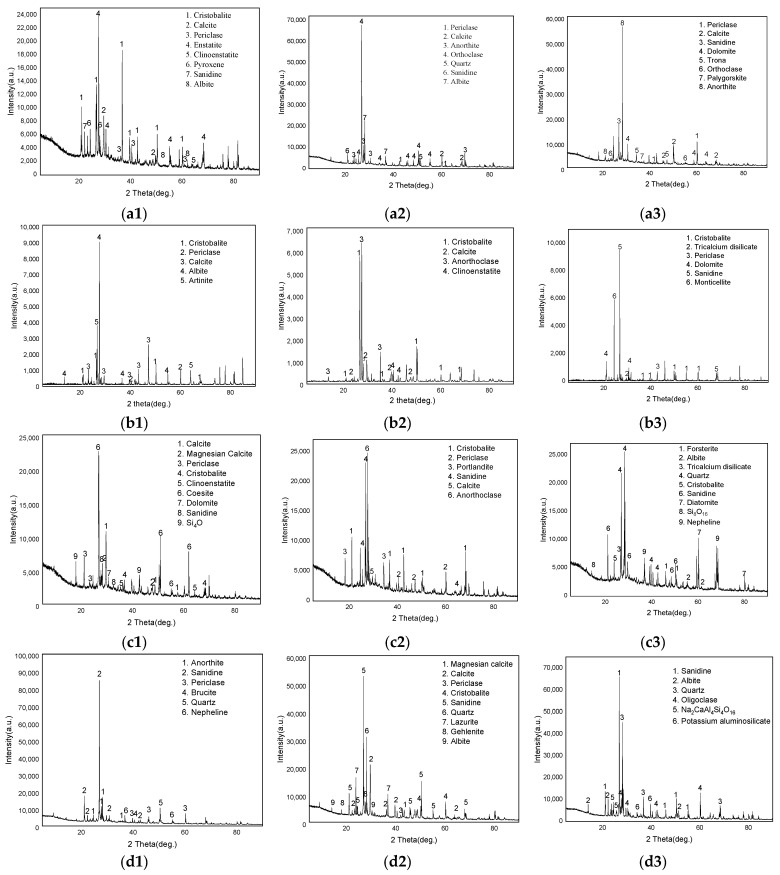
X-ray diffraction results: (**a1**–**a3**) UMD, (**b1**–**b3**) UAD, (**c1**–**c3**) UW, (**d1**–**d3**) DMD.

X-ray diffraction (XRD) phase analysis was conducted on the samples specified in the test plan, with results shown in Figure 12. The phase analysis indicates that the main concrete structures in the reservoir area share similar phase compositions. This suggests that identical construction materials were used during the early construction phase, with no differentiated designs implemented for different structures. Notably, diffraction peaks corresponding to magnesium sulfate crystals were detected in surface samples from the seaward face downstream of the main dam in Figure 12(b1), whereas these peaks were absent in other concrete sampling locations and in deeper seaward-facing layers. This observation suggests that the physical expansion resulting from salt crystallization due to marine salt weathering may contribute to the reduced density of the surface concrete on the seaward face.

To verify the aforementioned effects, tests were conducted on the water environment surrounding the main reservoir structure. The results of pH and corrosive ion content analyses for the environmental water and soil are presented in Table 4 and Table 5. As shown in the tables, the pH values of both reservoir water and accumulated water within the corridor were 6.79 and 6.73, respectively, indicating neutral to slightly acidic water quality. According to the provisions of Concrete Mixing Water (JGJ 63-2006) [25], the water environment of this reservoir meets the requirements and does not present acidic corrosion conditions. The measured chloride ion concentrations in the water samples were 13.39 mg/L and 14.36 mg/L, while the chloride ion concentration in soil samples ranged from 145.28 mg/L to 241.87 mg/L. Although below the specified standard, since the reservoir’s primary material is plain concrete, environmental chloride salts are not considered to cause electrochemical corrosion of the concrete. The measured sulfate ion concentrations in water samples were 9.04 mg/L and 15.78 mg/L, while chloride ion concentrations in soil samples ranged from 44.16 mg/kg to 237.75 mg/kg. These values fall below the requirements for sulfate environment action level IV-C specified in the Code for Durability Assessment of Hydraulic Concrete Structures (SL775-2018) [26]. Therefore, sulfate erosion damage is not considered for the main concrete structures of the dam. According to the provisions of the “Code for Durability Assessment of Hydraulic Concrete Structures” (SL 775-2018) [26], the reservoir’s carbonation environment category is I-C, corresponding to outdoor wet-dry alternating zones; The chloride environment category is II-D, corresponding to marine atmospheric zones. The sulfate environment category is below IV-C, meaning that in the reservoir’s external environment, hydration product deterioration caused by corrosive factors is attributed solely to atmospheric carbonation effects. In contrast, downstream areas, situated in a coastal saline weathering environment, exhibit salt crystallization expansion cracking, leading to severe surface strength degradation.

#### 3.3.2. Microstructural Analysis

**Figure 13:**
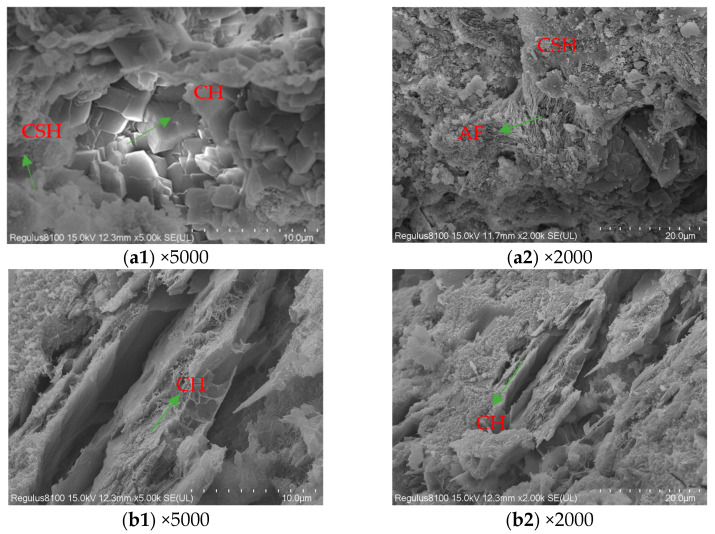
SEM images at different magnifications for different regions: (**a1**,**a2**)—surface layer, (**b1**,**b2**)—inner layer.

To further elucidate the erosion mechanisms in the downstream section, concrete samples were collected from different depths on the downstream face of the main dam, which exhibits the lowest water resistance, for microstructural analysis. Sampling locations included the surface layer and concrete 100 mm below the seepage point. Based on water resistance tests, this area exhibits high density and was used as an intact, undamaged reference. The results are presented in Figure 13.

Figure 13(a1) shows distinct cubic crystals formed within the internal pores of the sample. These crystals are stacked within the pores, with no other hydration products present internally. Irregularly shaped CSH gel is observed in multiple locations outside the fractures, suggesting these are CaCO_3_ crystals. Figure 13(a2) exhibits a distinct lamellar AFm structure with residual CSH gel particles within its matrix, though the presence of Al in the cementitious material cannot be ruled out. The surface structure shows numerous cavities with insufficient filling density. In contrast, the internal structure in Figure 13(b1,b2) exhibits distinct crystalline layered stacking and retains substantial hexagonal CH lamellar organization. This indicates that hydration products in the inner layer sample (100 mm depth from the downstream water-facing surface of the main dam) still preserve a large amount of CH crystals unaffected by carbonation erosion. These crystals form a compact layered or networked structure, ensuring the uniformity of the concrete’s internal structure.

**Figure 14:**
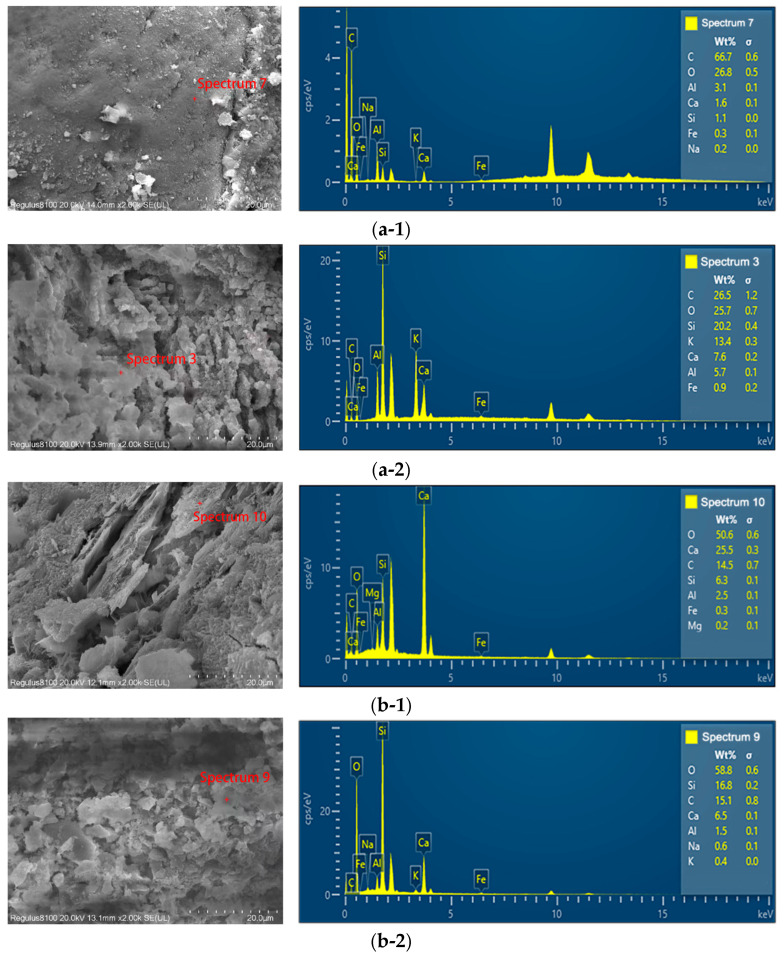
EDS spectrum element results: (**a-1**,**a-2**)—surface, (**b-1**,**b-2**)—subsurface.

Furthermore, EDS elemental analysis was conducted, with results shown in Figure 14. Samples a-1, a-2, b-1, and b-2 correspond to the surface crystalline structure, surface cementitious structure, inner crystalline structure, and inner cementitious structure, respectively. Regarding crystalline materials, the surface structure exhibits a high C content of 66.7%, followed by O and Al. This indicates that the cement hydration product CH has been fully converted to CaOH. The relatively high Al content in the surface layer is attributed to the presence of Aft cementitious materials. In contrast, the inner structure predominantly contains calcium and oxygen, suggesting a higher calcium hydroxide content with significantly reduced carbonation effects. Regarding the cementitious material, the surface layer contains calcium silicate hydrate alongside a notable increase in potassium (K) content. This may stem from coastal seawater, where chloride salts and other components infiltrate the concrete surface. The crystallization and expansion of these salt solutions within the concrete structure cause structural softening and increased porosity. In contrast, elemental concentrations within the internal structure remain consistently low. The impact of seawater on dam concrete does not extend beyond 100 mm.

## 4. Conclusions

Based on the analysis of the chlorine permeability resistance and micro-pore structure mechanisms in submerged dam concrete under long-term service, the main findings are summarized as follows:

(1) After more than 75 years of service, no water penetration was observed in the upstream section of the main dam (UMD) or the upstream section of the auxiliary dam (UAD) under a water pressure of 0.8 MPa for 24 h, whereas significant leakage occurred in the downstream section of the main dam (DMD).

(2) As the depth of water exposure increases, the penetration height in the concrete rises. The improvement in impermeability height from 0–50 mm to 50–100 mm can reach several times. This trend diminishes markedly beyond a depth of 100 mm. The penetration times for DMD were 0.5 h within 0–50 mm, 3.5 h within 50–100 mm, and more than 24 h beyond 100 mm.

(3) Mercury intrusion porosimetry (MIP) analysis indicates that the pore structure in DMD is coarser compared to other sections. The median pore diameter is 306.7 nm, and the average pore diameter is 55.4 nm. The pore content and porosity reach 35.64% and 3.961 mm, respectively.

(4) X-ray diffraction (XRD) and SEM analyses reveal that due to long-term exposure to seawater, sulfate ions and alkali metals in the downstream concrete react with the concrete matrix, leading to the formation of related salt crystals. Under the combined effect of prolonged carbonation, this results in increased porosity and reduced impermeability.

(5) Limitations of the study: This investigation is based on concrete samples from a single reservoir, and the findings may have limited applicability to other dams with different materials, construction practices, or environmental conditions. The study primarily focused on impermeability and pore structure, without considering reinforcement corrosion, micro-cracking, or dynamic flow effects. In addition, the pore characterization methods used may not fully capture pore connectivity or transport pathways. Future studies could be extended through multi-dam comparisons, long-term field monitoring, and advanced three-dimensional pore network analyses to further elucidate the long-term durability mechanisms of submerged concrete.

## Figures and Tables

**Table 1:** Mix proportions for typical dam sections.

Gradation	Cement	Fly ash	Water	Sand	Aggregate (5–20 mm)	Aggregate (20–40 mm)
Two-stage gradation	260	70	140	700	600	600
Three-stage gradation	240	60	145	600	800	800

**Table 2:** Relative permeability coefficient test results.

Engineering Section	Dept (mm)	Permeation Condition	Permeation Test Duration (h)	Permeation Height (mm)	Permeability Coefficient (cm/s)
UMD	0~50	No	24	0.6	<1.0 × 10^−13^
	50~100	No	24	0.4	<1.0 × 10^−13^
	100~150	No	24	0.3	<1.0 × 10^−13^
DMD	0~50	Yes	0.5	30.0	9.19 × 10^−9^
	50~100	Yes	3.5	30.0	1.31 × 10^−9^
	100~150	Yes	24	0.5	<1.0 × 10^−13^
UAD	0~50	No	24	0.3	<1.0 × 10^−13^
	50~100	No	24	0.2	<1.0 × 10^−13^
	100~150	No	24	0.1	<1.0 × 10^−13^
UW	0~50	No	24	14.6	4.54 × 10^−11^
	50~100	No	24	0.6	<1.0 × 10^−13^
	100~150	No	24	0.1	<1.0 × 10^−13^

**Table 3:** Test results for hardened bubble parameters.

Engineering Location	Test Sample Depth (mm)	Air Content (%)	Average Chord Length (mm)	Most Probable Chord Length (mm)	Bond-Air Ratio	Specific Surface Area (mm^2^ /mm^3^)	Porosity (mm^−1^)	Bubble Spacing Factor (mm)
UMD	0~50	4.88	0.070	0.0129	4.10	56.87	0.694	0.0721
	50~100	2.26	0.047	0.0118	8.85	85.27	0.481	0.0704
	100~150	2.86	0.084	0.0121	6.99	47.74	0.342	0.1131
DMD	0~50	35.64	0.090	0.0129	0.56	44.45	3.961	0.0126
	50~100	44.48	0.118	0.0138	0.45	34.03	3.784	0.0132
	100~150	16.45	0.094	0.0151	1.22	42.53	1.749	0.0286
UAD	0~50	2.24	0.048	0.0119	8.93	82.99	0.465	0.0726
	50~100	7.63	0.057	0.0125	2.62	69.80	1.331	0.0376
	100~150	15.68	0.072	0.0125	1.28	55.80	2.188	0.0229
UW	0~50	7.03	0.096	0.0150	2.84	41.46	0.729	0.0686
	50~100	13.87	0.087	0.0133	1.44	45.90	1.591	0.0314
	100~150	5.29	0.074	0.0150	3.78	54.18	0.716	0.0698

**Table 4:** pH and corrosive ion content of environmental water samples.

Water Sample	Sampling Location	pH	Sulfate Ion (mg/L)	Chloride Ion (mg/L)
1	Reservoir Area	6.79	15.78	14.36
2	Corridor	6.73	9.04	13.39

**Table 5:** Corrosive ion content in environmental soil samples.

Soil Sample	Sampling Location	Sulfate Ion (mg/kg)	Chloride Ion (mg/L)
1	Reservoir inlet	104.55	241.87
2	Reservoir mid-section	205.19	170.11
3	Dam perimeter	90.03	195.19
4	Downstream face (near the dam body)	44.16	145.63
5	Downstream face (far from dam body)	237.75	145.28

## Data Availability

The original contributions presented in this study are included in the article. Further inquiries can be directed to the corresponding authors.
